# Authoritarianism in youth social life: systematic literature review (2015–2025)

**DOI:** 10.3389/fpsyg.2026.1794108

**Published:** 2026-05-22

**Authors:** Gustavo Troncoso-Tejada, Karina Polanco-Levicán, José Luis Gálvez-Nieto, Eliana Ortiz-Velosa, David González Casas

**Affiliations:** 1 Programa de Doctorado en Ciencias Sociales, Universidad de La Frontera, Temuco, Chile; 2 Departamento de Trabajo Social y Servicios Sociales, Universidad Complutense de Madrid, Madrid, Spain; 3 Escuela de Psicología, Facultad de Ciencias Sociales y Educación, Universidad Santo Tomás, Temuco, Chile; 4 Departamento de Trabajo Social, Universidad de La Frontera, Temuco, Chile; 5 Facultad de Arquitectura Arte y Diseño, Universidad Católica de Temuco, Temuco, Chile

**Keywords:** authoritarianism, sexism, social dominance, systematic review, youth

## Abstract

**Introduction:**

In contexts of institutional erosion and polarization, authoritarian expressions among young people have emerged, straining democracy and civic coexistence. This review aims to identify the main manifestations of authoritarianism in youth social life and analyze their implications for democratic coexistence.

**Methods:**

A systematic review of the literature (2015-2025) was conducted in journals indexed in Web of Science and Scopus, following the PRISMA protocol. Out of n = 681 studies identified, n = 43 were selected for final analysis.

**Results:**

Four analytical dimensions were identified: social dominance, traditionalism-sexism-fundamentalism, mental health-parenting styles, and contextual-cultural perspectives, which expand the classical understanding of the phenomenon.

**Discussion:**

These dimensions highlight the increasing validation of authoritarian values linked to the rise of violence and social inequality, positioning authoritarianism as a relational and situated phenomenon that threatens democratic coexistence.

## 1 Introduction

Authoritarianism has been addressed in recent decades as a psychological and social phenomenon from a complex and nuanced analytical framework, articulating individual, cultural, and political dimensions. Various studies currently recognize it as a psychological construct of high relevance for the social sciences, reflecting the ways in which individuals negotiate tensions between order, security, and diversity (Lesgart, 2020; Moreno and Lagos, 2024; Verkuyten and Yogeeswaran, 2017; Rose, 2025). In recent years, findings have shown that certain authoritarian attitudes and behaviors tend to intensify in the face of perceived threats, whether from global crises such as the pandemic, economic instability that erodes social cohesion, or the increase in hostility toward minorities and socially marginalized groups (Osborne et al., 2023; Torres-Vega et al., 2021; Sochos, 2021; Da Costa Silva et al., 2024).

These contemporary manifestations of authoritarianism are linked to an increasing willingness to sanction dissenting ideas, as well as to a tendency to reaffirm one's own superiority or to demand submission in interactions among social groups (Conway et al., 2023; Costello et al., 2022; Duckitt, 2001). This dynamic highlights, given the conceptual

complexity involved, the need to specify how the expressions of authoritarianism are shaped and reproduced. While authoritarianism has been widely studied in reference to political regimes and systemic forms of domination, this review focuses on its conceptualization at the individual and relational level: as a psychological disposition, an ideological-political attitude, and a relational practice observable in everyday social life. This distinction is analytically relevant, as individual authoritarian orientations do not necessarily correspond to living under an authoritarian regime, but rather reflect ways in which people negotiate authority, hierarchy, and autonomy in their social interactions (Adorno et al., 1950; Altemeyer, 1981; Duckitt et al., 2010).

Since the mid-twentieth century, the understanding of authoritarianism has been shaped by analyses linking this phenomenon to the transformation of authority. Arendt (1996) argues that the historical decline of traditional authority has given rise to new forms of domination and contemporary authoritarian expressions, in which coercion and the restriction of individual freedoms constitute the core of these configurations. Complementarily, Weber (1964) maintains that authority and authoritarianism share a common anthropological basis related to power, differing mainly in their contextual and historical forms of expression: whereas legitimate authority is exercised through social recognition, authoritarianism is characterized by rigidity, control, and the unilateral imposition of will. Nevertheless, authority can be instrumentalized for authoritarian purposes, contributing to the consolidation of coercive forms of domination (Benbenaste et al., 2006).

During the 20th century, the study of authoritarianism gained particular relevance within social psychology. Adorno et al. (1950) linked the authoritarian personality to attitudes of obedience and anti-democratic social behaviors, while Altemeyer (1981) deepened the understanding of right-wing authoritarianism by connecting conservative values, submission to perceived legitimate authorities, and hostility toward outgroups. However, recent authors argue that these frameworks are insufficient to grasp current configurations of the phenomenon, which now include manifestations associated with both right- and left-wing ideological orientations, the consequences of social inequality and democratic institutional crisis, and their effects on mental and social health (Costello and Patrick, 2023; Costello et al., 2022; Bélanger et al., 2019; Troncoso-Tejada et al., 2025a; Moreno-Jiménez et al., 2013). This limitation calls for a theoretical update that accounts for new authoritarian expressions emerging in contemporary social dynamics (Deverson et al., 2025).

Building on these traditions as complementary analytical lenses, this review defines authoritarianism as a multidimensional construct encompassing three interconnected levels: psychological dispositions oriented toward cohesion and collective security at the expense of individual autonomy (Duckitt et al., 2010); ideological-political attitudes that legitimize hierarchies and restrict diversity; and relational practices of recognition and subordination observable in everyday domains such as education, activism, social media, and community interaction. Herzog (2021) captures this integrative dimension by showing how modern capitalism generates cultural abstractions that foster obedience and legitimize social hierarchies in daily life, configuring authoritarianism as a social syndrome that emerges particularly in contexts of crisis. Rather than adhering to a single theoretical tradition, this study regards these frameworks as complementary analytical lenses that shed light on different dimensions of a complex construct (Costello and Patrick, 2023), unified by a common thread: the legitimization of hierarchy, unilateral control, and the restriction of individual autonomy.

Youth constitutes a particularly relevant population for the study of authoritarian orientations. Adolescence and early adulthood are critical periods for the formation of political attitudes, identity, and social values, during which authoritarian dispositions emerge and consolidate through socialization processes shaped by family dynamics, peer relations, educational environments, and political crises (Yerofeyeva et al., 2024; Adorno et al., 1950; Altemeyer, 1981). Moreover, young people occupy a dual position in contemporary democratic life: as subjects exposed to authoritarian dynamics and as social actors whose orientations shape the future of democratic coexistence. Understanding how authoritarianism manifests in youth social life is therefore analytically relevant and politically urgent in contexts of institutional erosion and democratic polarization (Osborne et al., 2023; Levitsky and Ziblatt, 2018).

In the past decade, several studies in the social sciences have highlighted the emergence of authoritarian expressions linked to social discontent arising from processes of globalization and fractures in patterns of social coexistence (Ipar et al., 2024; Valencia, 2024; Borsuk and Levin, 2021). At the international level, economic, health, and religious crises have generated tensions that shape perceptions of authority and order, fueling feelings of insecurity and institutional distrust (Stiglitz, 2010), as illustrated by the case of democracy in Brazil and the fragmentation of the modern republic in Turkey (Tavares and da Silva, 2021; Yavuz, 2018).

Within this global context, multiple collective responses have been observed, among which social movements led by youth stand out as concrete expressions of these social grievances. Notable examples include the social upheavals in Chile in 2019 and Colombia in 2021 (Aguilar-Forero, 2022; Rivera-Aguilera et al., 2021), the global Feminist Movement (Alvarez and Navarrete, 2019), social responses to the COVID-19 pandemic in 2020 (Parra and Zorro, 2020), the Generation Z movement in Kenya in 2024 (Magack et al., 2025), diverse social reactions to the Gaza conflict, and the process of autocratization in Turkey under what has been termed a competitive authoritarian regime (Borsuk and Levin, 2021).

These expressions are particularly evident in regional and global contexts. In Latin America, the Latinobarómetro report (2024) documents growing disillusionment with democratic systems, increased distrust in public institutions, and rising preferences for authoritarian forms of government in response to perceptions of social and economic instability; a trend reinforced by far-right sectors that legitimize xenophobia and authoritarianism as means to promote perceptions of security, with violence and social inequality emerging as central factors in this citizen dissatisfaction (Dávila et al., 2020; López and Pereyra, 2021; Monsivais-Carrillo, 2020). Convergently, research conducted in the United States, United Kingdom, Egypt, Peru, and Chile demonstrates that social discontent and institutional distrust increase inclinations toward authoritarian behaviors and the resort to violence (Ramírez and Wood, 2023; Ghafar, 2021; Guzmán-Concha, 2023; Disi Pavlic et al., 2025), reinforcing the legitimation of social dominance among groups and the justification of violence as a means to address structural tensions (Cárdenas and Parra, 2010; Moreno-Jiménez et al., 2013; Bélanger et al., 2019; Moyano et al., 2022). Such expansion of authoritarianism across various spheres of social life constitutes a significant threat to the principles of civic coexistence, by normalizing violence and unilateral imposition as legitimate forms of conflict resolution (Levitsky and Ziblatt, 2018).

Given this growing complexity, the need arises to systematize the available evidence on the manifestations of authoritarianism among youth. Accordingly, this review addresses the question: What are the main manifestations of authoritarianism in youth social life according to the recent literature (2015–2025)?

This question encompasses multiple domains whose analytical coherence rests on a shared conceptual core: the tendency to legitimize hierarchies, restrict individual autonomy, and sustain unilateral forms of control across different spheres of youth social life (Arendt, 1996; Weber, 1964; Adorno et al., 1950; Duckitt et al., 2010). Following an inductive-deductive approach, emergent categories are allowed to expand the classical understanding of authoritarianism, provided they remain anchored to it as a social syndrome (Herzog, 2021).

The present article aims to identify areas of manifestation of authoritarianism among young people and analyze their implications for democratic social life. It is based on a systematic review of studies published in journals indexed in Web of Science and Scopus, following PRISMA criteria. This approach contributes to a more comprehensive and updated understanding of the phenomenon, particularly relevant in contexts of social and political tensions, and democratic ruptures where young people play an active role.

## 2 Method

A systematic literature review was conducted following the Preferred Reporting Items for Systematic Reviews and Meta-Analyses (PRISMA) protocol guidelines (Page et al., 2021). Table 1 presents the research question formulated according to PICOS criteria in the social sciences.

**Table 1:** Research question (PICOS).

PICOS component	Specification
Population (P)	Young people/adolescents (12–29 years old, aligned with UNESCO/WHO)
Intervention/Exposure (I)	Not applicable (descriptive review of manifestations)
Comparators (C)	Not applicable (non-comparative thematic synthesis)
Outcomes (O)	Areas of manifestation of authoritarianism in young people's social lives.
Study designs (S)	Quantitative, qualitative and mixed empirical studies indexed in WoS/Scopus (2015–2025).

The PICOS framework guided the definition of the eligibility criteria, specifying the population (youth aged 12–29), the phenomena of interest (manifestations of authoritarianism), and the inclusion of diverse study designs.

The question that guided the search strategy was: What are the main manifestations of authoritarianism in youth social life according to the recent literature (2015–2025)? The search was conducted in December 2025 in the Web of Science (WoS) and Scopus databases, selected for their scientific rigor and impact in the social sciences (Sánchez-Serrano et al., 2022).

The search strategy was based on English-language descriptors organized into three conceptual domains: 1. authoritarianism (authoritarianism, “social dominance orientation”, “authoritarian ideology/attitude/beliefs/conviction”, “antidemocratic/illiberal/nondemocratic/fascist ideology/attitude/beliefs/conviction”); 2. youth (youth, young people/population, adolescents, teen/teenagers); 3. perceptions (perception, assessment, appreciation, attitude).

The selection of descriptors followed an iterative consensus process among the five authors, informed by a preliminary review of seminal theoretical frameworks on authoritarianism (Adorno et al., 1950; Altemeyer, 1981; Duckitt et al., 2010) and by the terminological conventions used in previous systematic reviews on the topic. Terms were progressively refined through test searches to maximize retrieval sensitivity while maintaining thematic coherence.

These terms were combined following the structure: (authoritarianism terms) AND (youth terms) AND (perception terms), applying OR within each domain and AND between domains to ensure a comprehensive and systematic retrieval of studies across databases. The complete search strategies for each database are provided in Supplementary Material 1.

The search strategy was refined using the following filters: document type (original research articles and reviews), publication period (2015–2025), subject areas (social sciences, psychology, and political science), language (English, Spanish, and Portuguese), and open access. The inclusion of Spanish and Portuguese was a deliberate methodological decision aimed at broadening the geographical scope of the review beyond exclusively English-language literature, given that Latin America and other regional contexts constitute highly relevant settings for the study of youth authoritarianism, institutional distrust, and democratic erosion (Levitsky and Ziblatt, 2018; Latinobarómetro, 2024; Moreno and Lagos, 2024; Troncoso-Tejada et al., 2025b).

### 2.1 Eligibility criteria

Inclusion criteria: Scientific articles indexed in WoS/Scopus addressing authoritarian expressions or tendencies in adolescents and young adults. Studies included empirical research as well as review articles incorporating secondary data analysis. The focus was on attitudinal perceptions related to social cohesion, control, domination, or anti-democratic orientations (Arendt, 1996; Weber, 1964; Adorno et al., 1950), based on youth samples (12–29 years) and considering contextual factors such as family and culture. Eligible studies were published between 2015 and 2025, in English, Spanish, or Portuguese, and had accessible full text (open access).

The studies included samples of adolescents and young people aged 12–29 years, encompassing the transitions from adolescence through youth to early adulthood to capture psychological, social, and emotional development as it relates to authority and authoritarianism (Yerofeyeva et al., 2024).

This broad operational range aligns with flexible international definitions: UNESCO considers standard youth as 15–24 years but recognizes its culturally adaptable nature; the World Health Organization (WHO) defines early adolescence (10–14 years), youth (15–24 years), and young adults (up to 29 years). This breadth methodologically justifies the use of multiple search descriptors (“youth”, “young people”, “adolescents”, “teenagers”) given international terminological variability in the scientific literature.

Exclusion criteria: Studies on authoritarian/totalitarian regimes (with a systemic rather than an individual focus); studies without a youth population; studies outside thematic scope; theses, conference proceedings, or non-indexed publications (see Table 2 for full details).

**Table 2:** Eligibility, inclusion, and exclusion criteria.

Criterion	Inclusion criteria	Exclusion criteria
Type of study	Peer-reviewed articles indexed in Web of Science or Scopus, including empirical studies and review articles with empirical analysis.	Theses, conference proceedings, non-indexed publications, theoretical or conceptual papers without empirical data, editorials, letters to the editor, and opinion-based articles.
Temporality	2015–2025.	Studies outside this period.
Population	Adolescents and young people (12–29 years).	Studies without a youth population
Conceptual focus	Authoritarian attitudes in young people.	Studies focused exclusively on authoritarian or totalitarian political regimes at the systemic level, without an individual-level focus.
Scope	Social cohesion, control, domination, or anti-democratic orientations.	Studies outside the thematic scope
Language	English, Spanish, Portuguese	Other languages.
Subject area	Social sciences, psychology, political science.	Other areas.
Full text	Open access/accessible full text.	Studies without accessible full text.

### 2.2 Procedure

The study selection process followed the PRISMA 2020 framework, structured into four sequential phases: identification, screening, eligibility, and inclusion (Page et al., 2021). During the identification phase, records retrieved from the databases were compiled, and duplicate records were identified and removed using Excel tools.

In the screening phase, two independent reviewers assessed titles and abstracts to determine their relevance according to the predefined inclusion and exclusion criteria. In the eligibility phase, full-text articles were evaluated based on the PICOS criteria (Table 1). Disagreements at both screening and eligibility stages were resolved through discussion and consensus among the five authors.

**Figure 1:**
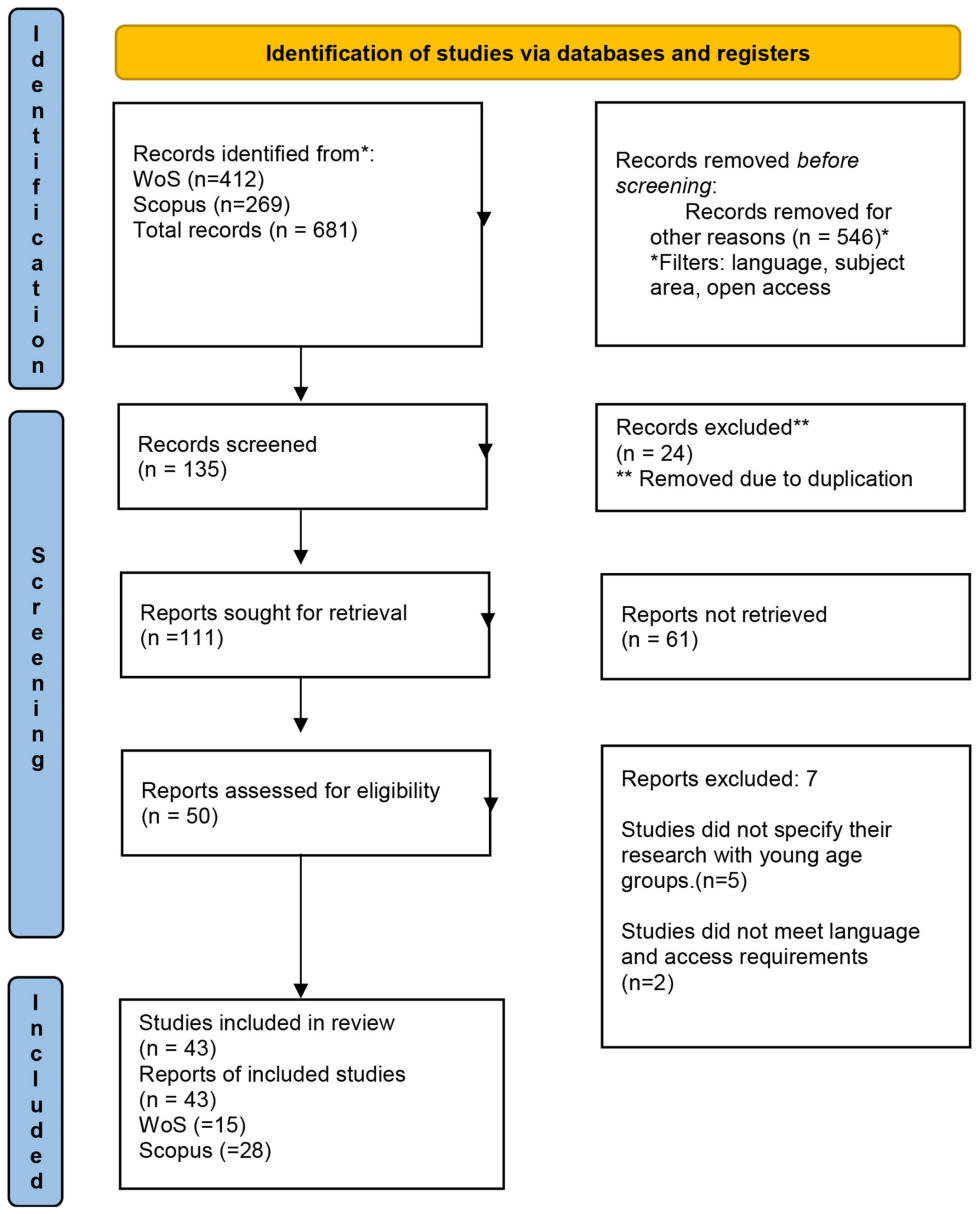
Flowchart of the systematic review (adapted from Page et al., 2021).

In the inclusion phase, studies meeting all eligibility criteria were retained for the final synthesis. The overall selection process is presented in the PRISMA flow diagram (Figure 1). Data extraction was systematically documented in Excel (Appendix—Supplementary Material 2: Data Extraction Database). No prior protocol registration was conducted.

### 2.3 Analysis strategy

The articles were analyzed using thematic content analysis with an inductive-deductive analytical approach (Bardin, 1991; Strauss, 1987; Taylor and Bogdan, 1987), considering an initial deductive categorization guided by theoretical frameworks regarding cohesion vs. autonomy and unilateral control (Arendt, 1996; Weber, 1964; Adorno et al., 1950). Additionally, methodological variables (design and instruments), participants' age range (12–29 years), and family, cultural, and social factors were considered.

The thematic categories emerged through a multi-stage process. Authors first reviewed titles, abstracts, keywords, discussions, and conclusions to gain an initial overview of the topics addressed. Full texts were then read in their entirety, and the main themes of each study were identified. Following repeated readings, inductive coding was conducted, emerging categories were identified, and studies were grouped by thematic affinity (Bardin, 1991), through iterative discussion and consensus among all five authors. Inter-coder agreement was not formally quantified; instead, coding decisions were validated through content triangulation (Denzin, 2017) and hermeneutic synthesis (Gadamer, 2004), identifying recurrent patterns, frequencies, convergences, and divergences across the reviewed studies. The complete coding scheme, including study type, main instruments, primary findings, and thematic category assigned to each of the n = 43 included studies, is documented in the Data Extraction Database (Supplementary Material 2).

Independently of the article selection process, methodological quality was assessed using JBI-specific checklists (see Supplementary Material 3) for each study type (Lockwood et al., 2015; Aromataris et al., 2015; McArthur et al., 2025). Three authors independently applied the checklists, classifying each item as “Yes”, “No”, “Unclear”, or “Not applicable”. Scores were calculated as the percentage of items rated affirmatively out of the total applicable items. Discrepancies were resolved through discussion with the other two authors until consensus was reached. No articles were excluded on the basis of risk of bias; however, the identified limitations were taken into account in the interpretation of the findings, with results tabulated by author, study type, objectives, country, region, and topic.

### 2.4 Ethical considerations

Ethical approval was not required, as this study involved published and openly accessible literature.

## 3 Results

The selection of articles followed consecutive stages according to the PRISMA flowchart (Figure 1). The initial search yielded *n* = 681 articles (Web of Science *n* = 412; Scopus *n* = 269). After applying filters (language, thematic area, open access), *n* = 135 eligible records were generated (Page et al., 2021). Subsequently, after removing duplicates (*n* = 24), *n* = 111 unique articles were recorded (WoS: *n* = 40; Scopus: *n* = 71). In the second stage, screening by titles, abstracts, and keywords, according to the objective and research question, excluded *n* = 61 articles, selecting *n* = 50 records for full-text reading. Finally, comprehensive review and strict application of eligibility criteria confirmed the sample of *n* = 43 articles for content analysis (WoS: *n* = 15; Scopus: *n* = 28).

### 3.1 General characteristics of the studies

The final sample comprised 43 empirical articles published between 2016 and 2025, identifying four coherent thematic categories of youth authoritarianism manifestation: (1) Social dominance (12 studies), (2) Traditionalism, sexism, and fundamentalism (11 studies), (3) Mental health and parenting styles (8 studies), and (4) Contextual and cultural perspectives (12 studies), according to the synthesis matrix (see Table 3). The classification considered explicit mentions of authoritarianism in young populations (12–29 years), with a concentration of studies in the last five years, particularly from 2020 onwards.

**Table 3:** Final selection matrix (studies included in the systematic review).

Year	Lead author	Country	Region	Study type	Age Range/Mean (years)	Topic	Category
2025	Weber, L.M.	Germany	Europe	Quantitative—Scales	M = 24.0 years.	Empathy and social dominance.	Social Dominance
2025	Daldrop, C.	United States	North America	Quantitative—Scales	Young leaders.	Youth leadership	Social Dominance
2024	Benjamin, S.	Finland	Europe	Mixed—Surveys.	16–19 years.	Social dominance.	Social Dominance.
2023	Grindal, M.	United States	North America	Quantitative—Scales	18–25 years (M = 21.7 years).	Racism and far-right attitudes.	Social Dominance.
2023	Daldrop, C.	United States	North America	Quantitative—Scales	M = 27.54 years.	Youth leadership.	Social Dominance.
2023	Travaglino, GA.	Italy	Europe	Quantitative—Scales	M = 16.70 years.	Masculine validation and criminal groups.	Social Dominance.
2022	Jylhä, KM.	Sweden	Europe	Quantitative—Scales	Young people.	Xenophobia.	Social Dominance
2022	Pauwels, LJR.	Belgium	Europe	Quantitative—Scales	M = 21.07 years.	Prejudice.	Social Dominance.
2022	Etchezahar, E.	Spain	Europe	Quantitative—Scales	M = 19.88 years.	Social dominance.	Social Dominance.
2021	Ilmarinen, VJ.	Multinational	Europe	Quantitative—Scales	Young people.	Anti-immigration attitudes and negative environmental values.	Social Dominance.
2019	Sprong, S.	Multinational	Europe, North America, South America, Asia, Oceania y Africa	Quantitative—Scales	M = 22.53 years.	Anomie, inequality, and strong leadership.	Social Dominance
2016	Derado, A.	Croatia	Europe	Mixed, scales, and structured and semi-structured interviews.	16–25 years.	Populism and strong leaders.	Social Dominance.
2025	Karasavva, V.	Canada	North America	Quantitative—Scales	M = 20.07 years/M = 21.13 years.	Racism, xenophobia, authoritarianism, minority violence, and sexism.	Traditionalism
2025	Zhang, JW., et al	United Kingdom	Europe	Quantitative—Scales	M = 26.2 years.	Sexism, violence, and hostility	Traditionalism
2024	González-Fuentes, JA.	Spain	Europe	Quantitative—Scales	M = 20.07 years.	Discrimination against sexual minorities.	Traditionalism
2022	Cinquegrana, V	Italy	Europe	Quantitative—Scales	M = 24 years.	Ambivalent sexism and psychological violence.	Traditionalism
2022	Off, G.	Multinational	Europe	Quantitative—Scales	18–29 years.	Modern sexism.	Traditionalism.
2022	Saroglou, V.	Multinational	Europe, Asia, Middle East y Latin America	Quantitative—Scales	M = 21.82 years.	Religiosity and fundamentalism.	Traditionalism.
2021	Rollero, C.	Italy	Europe	Quantitative—Scales	M = 23.09 years.	Sexisms and violence.	Traditionalism.
2020	Konopka, K.	Poland	Europe	Quantitative—Scales	M = 24.80 years/ M = 22.50 years/M = 29.35 years.	Transphobia, religiosity, and fundamentalism.	Traditionalism.
2019	Taufik, A.	Indonesia	Asia	Quantitative—Scales	Age not specified.	Fundamentalism, religiosity, and authoritarianism.	Traditionalism.
2018	Hannover, B.	Germany	Europe	Quantitative—Scales	M = 21.31 years.	Sexism, religiosity, and fundamentalism.	Traditionalism.
2018	Parent, M.C.	United States	North America	Quantitative—Scales	M = 20.10 years.	Transphobia and religious fundamentalism.	Traditionalism.
2025	Pinquart, M.	United States, Brazil, Spain	North America, South America, Europe	Quantitative—correlations	M = 15.08 years.	Authoritarian parenting and reduced substance use.	Mental health
2025	Pirzada, S. y Valadez, E.A.	United States	North America	Quantitative—Scales	M = 15.40 years.	Parenting style and SES impact on cognitive control.	Mental health
2025	Agudelo-Hernández, F	Colombia	Latin America	Cross-sectional quantitative—scales	Young people, age not specified.	Mental health importance and human rights.	Mental health
2024	Bobba, B.	Italy	Europe	Quantitative—Longitudinal	M = 15.61.	Family parenting and ethnic prejudice.	Mental health.
2023	Danilova, Y.	Russia	Eastern Europe	Quantitative—Scales	15-16 years.	Family parenting.	Mental health.
2023	Epstein, R.	United States	North America	Quantitative—Scales	M = 27.6 years.	Infantilisation, authoritarianism, and intolerance.	Mental health.
2018	Carlo, G.	United States	North America	Quantitative—Scales	M = 10.4 years (5th grade−12th grade).	Authoritarianism and prosocial behaviour.	Mental health.
2017	Slone, M.	Israel	Middle East	Quantitative—Scales	M = 12.94 years.	Parental styles and post-traumatic symptoms.	Mental health
2025	Stefanel, A.	Romania	Eastern Europe	Quantitative—nationally representative survey.	18–35 years.	Romanian youth and digital authoritarianism.	Context-Culture
2025	Majtényi, B.	Hungary	Central and Eastern Europe	Qualitative—Essay	University students.	Authoritarian control over universities.	Context-Culture
2024	López-Hornickel, N.	Chile, Colombia, Dominican Republic , México, Peru	Latin America	Quantitative—Scales ICCS	13-14 years (8th grade).	Authoritarianism and school gender equity.	Context-Culture
2023	Shamionov, RM.	Russia	Europe del Este	Quantitative—Scales	M = 21.8 years.	Authoritarianism, trust, and civic participation.	Context-Culture.
2022	Schäfer, A.	Multinational	Europe	Narrative	Young people.	Young generations and authoritarian populist voting.	Context-Culture.
2022	Prati, G.	Italy	Europe	Quantitative—Scales	M = 19.18 years.	Climate change and personality.	Context-Culture.
2022	Voces, C.	Spain	Europe	Quantitative—Secondary analysis of surveys—logistic regression models	Age categories.	Democratic erosion in Spanish youth/Pandemic, democratic support, and authoritarianism.	Context-Culture.
2022	Chiru, M.	Multinational	América Latina y Europe	Quantitative—Comparative through surveys/scales	M = 18–34 years.	Technocracy, authoritarianism and democracy.	Context-Culture.
2020	Krämer, M.	Namibia	Africa	Qualitative—Ethnographic	Young people.	Youth vs. neo-traditional authority.	Context-Culture.
2020	Russo, S.	Italy	Europe	Experimental quantitative—Scales	M = 29.70 years.	Threat overestimation and authoritarianism.	Context-Culture.
2020	Abbott, P.	Multinational	Middle East and North Africa	Quantitative comparative surveys	18–34 years.	Youth, Arab Spring participation, and social justice.	Context-Culture.
2017	Méndez, I.	Spain	Europe	Quantitative—Scales	M = 14.41 years.	Risk factors: school bullying and authoritarian parenting style.	Context-Culture.

Although these categories share a common conceptual core, the legitimization of hierarchies, unilateral control, and restriction of autonomy, their distinction responds to complementary analytical criteria rather than mutually exclusive domains (Costello and Patrick, 2023). Specifically, sexism was treated as a separate category from social dominance because the reviewed studies address it in relation to gender hierarchies, religious fundamentalism, and intimate partner violence, constituting a normative repertoire beyond general intergroup dominance orientation. Similarly, authoritarian parenting styles were distinguished as a separate dimension because the studies link them specifically to psychological wellbeing trajectories, emotional regulation, and the development of authoritarian dispositions in youth, rather than treating them solely as socialization mechanisms.

The studies were mainly conducted in Europe (51%, *n* = 22) (Germany, Finland, Italy, Sweden, Belgium, Spain, Poland, Croatia, United Kingdom, Hungary, Romania, Russia), North America (19%, *n* = 8) (United States, Canada), Middle East (2%, *n* = 1) (Israel), Africa (2%, *n* = 1) (Namibia), Asia (2%, *n* = 1) (Indonesia) and Latin America (2%, *n* = 1) (Colombia). In addition, 21% (*n* = 9) correspond to multinational studies covering Europe, North America, Latin America, Middle East, Asia and Africa. This demonstrates that research on authoritarianism in youth social life is concentrated in Global North contexts, with a more limited but analytically relevant presence of Global South countries in multicenter designs.

Regarding methodological design, quantitative approaches predominate (*n* = 39, 91%), employing psychometric scales (mostly cross-sectional studies, *n* = 37, 86%), complemented by a smaller number of mixed-methods studies (*n* = 2; surveys and interviews) and structured or semi-structured qualitative approaches (*n* = 2), particularly in research on strong leadership, populism, and youth political cultures. The themes covered include, among others, social dominance, racism, xenophobia, prejudice, sexism and violence, religiosity and fundamentalism, transphobia, parenting styles, mental health, civic participation, and democratic erosion (Table 3 for full details). These themes were subsequently grouped into second-order analytical categories based on conceptual affinity and level of analysis, through a process of discussion and consensus within the research team, resulting in four central categories: Social dominance (*n* = 12), Traditionalism, sexism and fundamentalism (*n* = 11), Mental health and parenting styles (*n* = 8), and Contextual and cultural perspectives (*n* = 12).

### 3.2 Risk of bias assessment

The application of JBI-specific checklists by study type revealed high overall methodological quality (mean 85%, range 63–100%). Of the 43 included articles, 40 were classified as high quality and 3 as moderate quality (Ilmarinen et al., 2021; Epstein et al., 2023; Schäfer, 2022). Cross-sectional studies predominate (86%, mean 8/8 = 89%) with strengths in validated scales (social dominance/traditionalism) and statistical analysis, but weaknesses in confounding factors (45%). Qualitative (5%, mean 9/10 = 90%) and narrative (2%, 83%) studies are excellent for deepening reflection on authoritarianism in the social lives of young people. The limitations identified in studies with moderate scores were considered with caution during the interpretive synthesis, without compromising their inclusion. No articles were excluded due to risk of bias.

### 3.3 Social dominance

The first identified area corresponds to social dominance, understood as the tendency to legitimize hierarchies between groups and to justify the superiority of certain collectives over others, thereby enabling authoritarian dispositions and practices in youth social life. This category includes 12 studies, predominantly quantitative and scale-based, conducted in Europe, North America, and multinational contexts, demonstrating how social dominance intertwines with prejudice, validation of strong leadership, and forms of symbolic and physical violence among young people.

Regarding intergroup relations, Benjamin et al. (2024) identify three youth profiles among vocational students in Finland, where 11% oriented toward exclusion exhibit high social dominance, dogmatism, and threat perception toward immigrants and Islam, justified by an exclusionary Finnish identity. Complementarily, Grindal and Haltinner (2023) and Jylhä et al. (2022) demonstrate in the United States and Sweden that social dominance articulates with racism, xenophobia, and support for far-right agendas, driven by threat perceptions regarding minorities and migrants. Pauwels and Williamson (2024) confirm in Belgium that high social dominance scores predict persistent prejudice, even controlling for sociopolitical variables, while Etchezahar et al. (2022) in Spain link this orientation to anti-egalitarian dispositions that legitimize hierarchies in youth social life.

Several studies underscore the connection between social dominance and the validation of strong leadership and authoritarian solutions. In this regard, the multinational analysis by Sprong et al. (2019), encompassing Europe, North America, South America, Asia, Oceania, and Africa, demonstrates that perceived economic inequality increases social anomie and the desire for strong leaders willing to employ non-democratic means, creating fertile ground for authoritarian expressions among youth. In Croatia, Derado et al. (2016) observe that youth sectors are willing to question or reject dimensions of the liberal representative democratic system in favor of populist leaderships perceived as more effective, complicating the relationship between social discontent, dominance, and authoritarianism. Convergent with these findings, the European multinational study by Ilmarinen et al. (2021) indicates that social dominance intertwines with anti-immigration attitudes and rejection of environmental agendas, articulating identity and nationalist concerns that seek to reorder social coexistence from hierarchical positions.

Another aspect links social dominance to the construction of status and honor in youth social life. For instance, in Italy, Travaglino et al. (2023) demonstrate how the validation of masculine honor and belonging to criminal groups associated with social dominance, insofar as youth come to legitimize criminal organizations as spaces of recognition and power in contexts of exclusion and inequality. This logic of pursuing status through aggression and control reinforces authoritarian patterns in everyday interactions, where the exercise of force becomes naturalized as a legitimate pathway for social positioning.

Finally, some studies address social dominance from the dynamics of leadership and interpersonal relationships. The works of (Daldrop et al. 2023, 2025) in the United States reveal that youth perceptions of leadership are permeated by biases combining dominance, authority, and distrust, particularly toward young figures, potentially reproducing vertical and undemocratic leadership styles in educational and community settings. For their part, Weber et al. (2025) in Germany explore the relationship between empathy and social dominance, demonstrating that lower empathy levels are associated with greater acceptance of hierarchies and authoritarian dispositions in peer relationships.

### 3.4 Traditionalism, sexism and fundamentalism

The second identified area corresponds to traditionalism, sexism, and fundamentalism, understood as belief systems that serve to legitimize gender and sexual hierarchies, naturalize inequality between groups, and sustain rigid views of social order among youth. This category includes 11 quantitative studies based on scales, primarily conducted in Europe and North America, demonstrating how these orientations intertwine with authoritarianism, prejudice, and validation of symbolic and material violence toward women and sexual minorities.

Regarding sexism and gender violence, Rollero et al. (2021) in Italy demonstrate that youth exhibiting higher levels of social dominance and sexist attitudes tend to minimize psychological violence and restrictions on women's freedom, legitimizing forms of control and emotional abuse as “normal” behaviors in gender relations. Complementarily, Cinquegrana et al. (2022) show that ambivalent sexism links to acceptance of psychological violence in youth dating contexts, contributing to the reinforcement of relationships based on female subordination and idealization of traditional roles. Within this framework, Zhang et al. (2025) in the United Kingdom position hostile sexism as a predictor of support for overtly violent youth behaviors toward women, expressing explicit forms of gender authoritarianism.

Studies on modern sexism and meritocratic logics delve deeper into how expressions of intolerance are reconfigured. In this regard, Off et al. (2022), in a European multinational study, identify among young men narratives that perceive advances in the rights of women and minorities as threats to their opportunities, articulating a modern sexism that rejects equality policies. This form links to ideologies that deny structural discrimination but justify status and opportunity differences, subtly reinforcing gender hierarchies.

Regarding sexual and gender minorities, the works of González-Fuentes et al. (2024) in Spain and Konopka et al. (2020) in Poland demonstrate that conservative religiosity, fundamentalism, and authoritarian orientations relate to higher levels of discrimination toward sexual minorities and transphobic attitudes among youth. While González-Fuentes et al. (2024) document rejection and exclusion toward LGB people in contexts discussing equality rights, Konopka et al. (2020) show that even in countries with relatively protective legal frameworks, high levels of prejudice toward trans people persist, sustained by traditional beliefs about gender and sexuality. In the United States, Parent and Silva (2018) find that youth transphobia associated with religious fundamentalism increases the likelihood of opposing policies favoring sexual minority equality, directly connecting these beliefs to political decisions affecting democratic coexistence.

Studies on religiosity, fundamentalism, and authoritarianism further expand this picture. Hannover et al. (2018) in Germany show that, among Christian and Muslim youth, the combination of high religiosity, fundamentalism, and authoritarian attitudes is associated with higher levels of ambivalent sexism, particularly among Muslim boys, where honor-based beliefs legitimize female subordination under a veneer of protection. In a comparative multinational perspective, Saroglou et al. (2022) demonstrate that religious fundamentalism, beyond political affiliation, is linked to dogmatism, rejection of diversity, and support for traditional hierarchies, reinforcing authoritarian dispositions among youth across different regions of the world. In Indonesia, Taufik and Farnanda (2019) shows that fundamentalism and religiosity intertwine with right-wing authoritarianism, configuring a framework in which obedience, submission to authority, and defense of traditional norms are projected onto hostile attitudes toward those who question the established order.

Finally, traditionalism is also articulated with racism, xenophobia, and intergroup violence, broadening the focus beyond gender. Karasavva et al. (2025) in Canada demonstrate that youth with high levels of authoritarianism, racism, and xenophobia show greater acceptance of violence toward minorities, including sexist expressions, suggesting a pattern of defending the traditional order against groups perceived as threatening.

### 3.5 Mental health and parenting styles

The third identified area corresponds to mental health and parenting styles, understood as the intersection between authoritarian parental practices and their impacts on psychological wellbeing, emotional regulation, and social behaviors among youth. This category includes 8 quantitative studies, predominantly scale-based, conducted in North America, Europe and Latin America, demonstrating how authoritarian parenting shapes trajectories toward authoritarianism in youth social life through mechanisms of control, infantilization, and emotional dysregulation.

Regarding parenting styles and mental health, Slone and Shoshani (2017) in Israel demonstrate that authoritarian parenting styles are associated with elevated posttraumatic symptoms among youth exposed to trauma, where rigid control exacerbates psychological vulnerability to adversities. Other studies reveal contextual effects on prosocial behaviors. For example, Carlo et al. (2018) in the United States find that, among Mexican-origin youth, maternal authoritarian parenting (high warmth + demandingness) predicts greater prosocial behaviors and better academic performance via self-efficacy, surpassing permissive or disengaged styles, although paternal effects are less consistent. This culturally adaptive dynamic contrasts with Danilova et al. (2023) in Russia, where orphaned adolescents perceive greater authoritarianism and control from institutional caregivers, associating with lower acceptance, empathy, and democratic orientation, fostering distrust and rejection of social cooperation.

Another axis links infantilisation and prejudice with mental health. Epstein et al. (2023) in the United States demonstrate that authoritarian parenting promotes infantilization in young adults, correlated with intolerance, authoritarianism, and reduced relational autonomy, hindering positive interpersonal bonds and reproducing hierarchical dynamics. In Italy, Bobba et al. (2024) document longitudinally that authoritarian family practices predict persistent ethnic prejudice in adolescents, mediating effects on psychological wellbeing through cognitive rigidity and social exclusion.

Finally, recent research addresses parenting, cognition, and human rights. Pirzada and Valadez (2025) in the United States show that authoritarian styles moderate the impact of socioeconomic status on child cognitive control, where parental rigidity limits executive development in disadvantaged contexts. Pinquart and Lauk (2025) in the United States, Brazil, and Spain confirm that authoritarian parenting reduces substance use among adolescents, suggesting adaptive benefits in behavioral risk prevention. Agudelo-Hernández et al. (2025) in Colombia emphasize authoritarian parenting as a barrier to the recognition of mental health as a human right, complicating its manifestation among youth by naturalizing control over emotional autonomy.

### 3.6 Contextual and cultural perspectives

The fourth identified area corresponds to contextual and cultural perspectives, which examine how macro-social, political, and cultural factors modulate manifestations of authoritarianism in youth social life. This category includes 12 studies (quantitative and qualitative) conducted in Europe, Latin America, Africa, and the Middle East, revealing how economic crises, digital polarization, democratic erosion, and cultural diversity complicate youth authoritarianism amidst ongoing global democratic ruptures.

Regarding democratic erosion and technocracy, Chiru and Enyedi (2022) demonstrate in nine European and Latin American countries that youth with high perceptions of political corruption prefer technocrats over elected leaders, associating with authoritarian attitudes that question democratic representativeness. In Spain, Voces and López (2022) document that the pandemic exacerbated youth democratic disaffection, increasing support for authoritarian governments among those perceiving freedom restrictions, particularly on the far right. Complementarily, Schäfer (2022) analyses European data confirming that younger generations vote for authoritarian populist movements, driven by systemic distrust rather than traditional ideology.

Studies on digital authoritarianism and civic participation highlight contemporary dynamics. In this regard, Ştefănel (2025) in Romania identifies that youth exposed to social media display tendencies toward digital authoritarianism, legitimizing online control and uniformity, although digital activism fosters antagonism against hierarchical structures. Shaminov et al. (2023) demonstrate in Russia that youth authoritarianism correlates inversely with trust and civic participation, but positively with digital leisure that reinforces social uniformity, suggesting that authoritarian youth tend to withdraw from civic action but consume passive social media that homogenize their beliefs and strengthen obedience.

In contexts of ideological polarization and academic control, Majtényi and Ryder (2025) qualitatively analyze in Hungary how young academics internalize obedience in response to systemic pressures that erode their critical autonomy. Russo et al. (2020) experimentally demonstrate in Italy that the overestimation of threats (migration, security) predicts youth authoritarianism, amplifying prejudices in contexts of economic and democratic uncertainty.

In Namibia, Krämer (2020) documents ethnographically youth resistance against corporatized neo-traditional authorities, where generational clashes complicate authoritarian loyalties in hybrid structures. Abbott et al. (2020) reveal in Arab countries that youth protests prioritize economy and corruption over human rights, challenging liberal narratives and evidencing pragmatic authoritarianism.

In Latin America, López-Hornickel et al. (2024) in five countries find that school youth authoritarianism associates with gender inequity, where rigid educational contexts reproduce hierarchies in everyday interactions. In Italy, Prati et al. (2022) show that youth climate concern increases with age and migrant tolerance, but decreases with nationalism and authoritarianism, positioning climate change as an antidote to ideological rigidities. Finally, Méndez et al. (2017) in Spain link authoritarian parenting styles with school bullying, contextualizing authoritarianism as a relational risk in educational settings.

## 4 Discussion

The findings of this review, drawn primarily from European and North American contexts, suggest that authoritarianism in youth social life manifests through four major and interrelated analytical axes: social dominance, traditionalism, sexism and fundamentalism, mental health and parenting styles, and contextual and cultural perspectives. Rather than functioning as isolated themes, these axes appear as a continuum of manifestations that connect dispositions, attitudes, and relational practices across different social contexts. Collectively, these results confirm the need to broaden the observational lens from a relatively stable individual disposition toward a network of practices, beliefs, and contexts that reconfigure hierarchies and forms of violence in strained democratic settings, consistent with approaches conceiving authoritarianism as a social syndrome emerging in contexts of crisis and structural discontent.

Overall, the studies reviewed, concentrated in Europe and North America, suggest that youth authoritarianism, at least in these contexts, takes diverse forms but shares a common underlying logic: the legitimization of hierarchies and the restriction of autonomy. Variation across domains does not imply separate phenomena, but rather different forms of expression depending on relational, institutional, and cultural context.

In the social dominance category, the reviewed studies indicate that intergroup hierarchies lie at the centre of youth authoritarian manifestations, consistent with Adorno et al. (1950), Adorno et al. (2006) postulates regarding orientation toward strong groups and hostility toward those perceived as inferior. Profiles such as the exclusion-oriented group described by Benjamin et al. (2024) rearticulate the notion of authoritarian personality anchored in group superiority, but situated today within frameworks of nationalism and identity conflicts surrounding migration and national belonging. Convergent with these findings, the works of Grindal and Haltinner (2023), Jylhä et al. (2022), Pauwels and Williamson (2024), and Etchezahar et al. (2022) demonstrate that social dominance links with racism, xenophobia, persistent prejudice, and anti-egalitarian dispositions, confirming that ethnic and racial hierarchies remain a privileged core for authoritarian expression among youth, as anticipated by social dominance orientation and right-wing authoritarianism models (Moreno-Jiménez et al., 2013; Moyano et al., 2022; Altemeyer, 1981; Cárdenas and Parra, 2010).

Another contribution from these studies demonstrates that social dominance articulates with support for strong leadership and non-democratic solutions, a central aspect in the classical readings of Arendt (1996) and contemporary threat management models of Duckitt (2001). In this regard, Sprong et al. (2019) show that perceived economic inequality fuels social anomie and the desire for leaders willing to employ non-democratic means, while Derado et al. (2016) and Ilmarinen et al. (2021) document that youth sectors remain open to questioning liberal democracy in favor of populist leaderships and anti-immigration and anti-environmental agendas. This convergence suggests that social dominance operates as a bridge between the subjective experience of structural discontent and the legitimization of authoritarian responses, reinforcing interpretations linking inequality, threat perception, and demand for strong order in contemporary democracies.

Furthermore, the connection between dominance, status, and violence highlights dimensions consistent with Weberian notions of power (Weber, 1964) and Arendt (1996) reflections on the substitution of authority by coercive forms. The works of Travaglino et al. (2023) and Daldrop et al. (2023, 2025) demonstrate that the pursuit of masculine honor, belonging to criminal groups, and youth leadership styles configure privileged scenarios for the normalization of force, control, and relational verticality. Additionally, Weber et al. (2025) provide evidence on the modulating role of empathy. In this respect, lower empathy levels are associated with greater acceptance of hierarchies and authoritarian dispositions. These findings engage with perspectives conceiving authoritarianism not only as adherence to order values but also as a relational style legitimizing humiliation and violence in everyday interaction. In this sense, the studies reviewed converge in showing that the core of this category is group superiority and vertical control as ways of organizing social coexistence among young people.

The studies on traditionalism, sexism, and fundamentalism demonstrate that gender and sexual hierarchies constitute a central axis for updating authoritarian repertoires among youth. The ambivalent and hostile sexism documented by Rollero et al. (2021), Cinquegrana et al. (2022), and Zhang et al. (2025) contributes to minimizing and normalizing psychological violence and restrictions on women's freedom, presenting forms of control, emotional abuse, and explicit aggression in gender relations as acceptable. In Weberian terms (1964), these relationships are sustained through unilateral imposition of will under the guise of legitimate traditional norms, limiting democratic plurality. In turn, research on modern sexism and meritocratic logic (Off et al., 2022) shows that some segments of youth shape intolerance through discourses of neutrality and merit, integrating traditional gender norms into a broader hierarchical repertoire that justifies inequalities as individual outcomes. These findings challenge the egalitarian assumptions of contemporary democracies and engage in research that, beyond the distinction between right-wing and left-wing authoritarianism (Bélanger et al., 2019; Costello et al., 2022; Costello and Patrick, 2023; Troncoso-Tejada et al., 2025a), warn how the defense of hierarchical moral orders can erode advances in equality policies and democratic coexistence.

Regarding sexual and gender minorities, the works of González-Fuentes et al. (2024), Konopka et al. (2020), and Parent and Silva (2018) demonstrate that conservative religiosity, fundamentalism, and authoritarian orientations translate into elevated levels of discrimination, transphobia, and opposition to equality policies. The literature on religiosity and fundamentalism (Hannover et al., 2018; Saroglou et al., 2022; Taufik and Farnanda, 2019) reinforces the above, showing that the combination of religiosity, honor-based beliefs, and political-religious dogmatism associated with ambivalent sexism, rejection of diversity, and support for traditional hierarchies. Collectively, these studies suggest that traditionalism, sexism, and fundamentalism function as normative matrices sustaining simultaneously gender, sexual, and ethnic hierarchies, articulating fertile ground for the expression of youth authoritarianisms in moral and religious terms. At the same time, although their expressions vary between ambivalent sexism, rejection of sexual minorities, religious dogmatism, and meritocratic discourse, all these repertoires converge in the legitimization of traditional hierarchies and the naturalization of inequality as a moral order.

The results concerning mental health and parenting styles underscore the importance of early socialization in shaping authoritarian dispositions. For example, the study by Slone and Shoshani (2017) demonstrates that authoritarian parenting styles are associated with post-traumatic symptoms, internal and external emotional dysregulation, and greater difficulties in affective management. This suggests that authoritarian parenting not only affects psychological wellbeing but also limits youths' capacity to process conflicts and disagreements without resorting to logics of control and submission.

However, the evidence also reveals that the effects of authoritarian parenting are not unequivocal but depend on how it combines with other relational dimensions and the context in which it is exercised. Carlo et al. (2018) demonstrate that, among Mexican-origin youth in the United States, maternal authoritarian parenting that is warm associates with greater prosocial behaviors and better academic performance, whereas less involved styles predict reduced prosociality. In contrast, Danilova et al. (2023) find that, among Russian orphaned adolescents, authoritarian control from institutional caregivers links with low acceptance, reduced empathy, and rejection of democratic values. These findings indicate that rigid authority can acquire different meanings: on one hand, in contexts of high warmth and family bonding, it may be interpreted as protective discipline; on the other, in vulnerability and institutional care settings, it is experienced as coercive control, reinforcing distrust and anti-democratic disposition.

Other studies expand this perspective by connecting authoritarian parenting with infantilisation, prejudice, and cognitive development (Epstein et al., 2023; Bobba et al., 2024; Pirzada and Valadez, 2025; Agudelo-Hernández et al., 2025). Collectively, these results suggest that authoritarian socialisation does not produce pathology alone but configures a continuum of relational responses spanning mental health, emotional regulation, prosociality and political attitudes. However, the meaning of this authority varies according to the context and relationship in which it is exercised. While in contexts of vulnerability and institutional care it tends to operate as coercive control (Benbenaste et al., 2006), in some family contexts marked by high warmth it may be interpreted as protective discipline. In both cases, authoritarian parenting contributes to shaping youth dispositions that can extend into broader social life through forms of control, submission, and restriction of autonomy.

Youth authoritarian manifestations cannot be understood apart from recent macro-political processes and cultural transformations, which, as noted at the beginning of this article, intensify threat perceptions and erode social cohesion, generating demands for coercive order at the expense of individual autonomy (Arendt, 1996; Weber, 1964). Studies on democratic erosion and technocracy (Chiru and Enyedi, 2022; Voces and López, 2022; Schäfer, 2022) demonstrate that disaffection towards parties, perceptions of corruption, and crisis experiences such as the pandemic fuel youth support for technocratic solutions and authoritarian populisms, suggesting the need to broaden the observational lens beyond authoritarian personality studies (Adorno et al., 1950) into contexts of institutional distrust. These dynamics are inscribed within a broader scenario of representation crisis and liberal democracy fatigue, where youth oscillate between disenchantment and demands for effective order, threatening Weberian plurality.

In the digital realm, the works of Stefanel (2025) and Shaminov et al. (2023) demonstrate that social media constitute an ambivalent space which, following Herzog (2021), may reproduce relational forms of authority and obedience. In this regard, on the one hand it fosters digital authoritarianism based on control, uniformity, and passive consumption of content aligned with official narratives; on the other, it promotes forms of activism challenging hierarchical structures and broadening political conflict. The negative correlation between youth authoritarianism and civic participation, alongside the positive association with digital leisure reinforcing ideological uniformity, suggests youth profiles withdrawing from the public sphere while immersing in homogeneous informational environments, legitimizing submission to perceived legitimate authorities (Altemeyer, 1981). This digital dynamic complicates authoritarianism as a social syndrome emerging in crises, diverting aggression toward dissent and normalizing hierarchies in everyday interactions.

Studies on polarization, perceived threats, and political cultures further complicate the observation of youth and authoritarianism, configuring as a social syndrome emerging in crisis contexts, integrating relational dimensions of obedience and sanctioning of dissent (Duckitt et al., 2010; Herzog, 2021). Majtényi and Ryder (2025) document how authoritarian control over universities erodes young academics' critical autonomy. Russo et al. (2020) demonstrate that overestimation of security threats fuels authoritarian attitudes, while Krämer (2020) and Abbott et al. (2020) show that, in African and Arab contexts, youth resistances and protests structure more around economy and corruption than explicit human rights demands. Finally, works such as López-Hornickel et al. (2024), Prati et al. (2022), and Méndez et al. (2017) underscore the role of educational institutions and global concerns like climate change in shaping authoritarian orientations, evidencing that schools and environmental debates constitute central spaces where hierarchies, inequities, and democratic visions are contested, threatening plurality through unilateral impositions (Weber, 1964; Arendt, 1996).

In short, the findings suggest that youth authoritarian expressions take their most clearly contextual form in this block. Authoritarianism appears here as a situated response to democratic erosion, crises of representation, digital polarization, and cultural transformation, conditions that intensify the demand for coercive order and weaken critical autonomy. Schools, universities, social media, and debates around climate change thus emerge as decisive spaces in which hierarchies, obedience, and democratic plurality are contested.

Overall, and on the basis of evidence predominantly from the Global North, this review suggests that youth authoritarianism is expressed as a multilevel continuum rather than as a set of isolated topics, though this pattern requires validation in Global South contexts. Although the findings take diverse forms depending on the domain analyzed, they all point to a common core of hierarchy legitimation, unilateral control, and restriction of autonomy, which is activated across intergroup, moral, relational, and contextual levels. Differences across studies do not undermine this convergence; rather, they show how authoritarianism adopts specific configurations according to the social, institutional, and cultural conditions in which it is embedded.

## 5 Limitations

This systematic review presents limitations in its design and scope. First, study selection was restricted to the descriptor “youth” (and its synonyms). In this regard, future studies are recommended to complement this with alternative terminology (e.g., “adolescents”, “emerging adults”) to broaden representativeness according to early age transitions.

Second, the restriction to Web of Science and Scopus introduces a structural limitation that deserves careful consideration. Although the search strategy deliberately included three languages —English, Spanish, and Portuguese— with the explicit aim of mitigating the Global North bias that characterizes systematic reviews restricted to English-language sources, the selection of these databases partially offsets this effort. Both WoS and Scopus systematically over represent journals from European and North American institutions, regardless of the language in which articles are written. This explains why 51% of the included studies originate from Europe and 19% from North America, while regions such as Latin America, Africa, and Asia appear only through multinational designs. This is not primarily a linguistic limitation, but a structural one rooted in the indexing logic of these databases, which grants lower visibility to scientific production circulating through regional platforms such as SciELO, Redalyc, or Dialnet. Additionally, the open-access criterion, adopted to ensure full-text accessibility during screening and coding as well as procedural transparency and replicability, may have further excluded relevant subscription-based studies, introducing a selection bias independent of study quality. As a consequence, relevant research on youth authoritarianism conducted in Global South contexts—where processes of democratic erosion, inequality, and institutional distrust are particularly acute—may have been systematically underrepresented. Future reviews should incorporate regional databases and subscription-based sources to better capture the geographical diversity of global research on this phenomenon.

In addition, although , the restriction to Web of Science and Scopus (open access, indexed) ensures rigour but excludes regional databases (SciELO, Redalyc, Dialnet) and others (Google Scholar). Whilst the search strategy included three languages, english, spanish, and portuguese, which partially broadens the geographical scope beyond exclusively English-language reviews, both WoS and Scopus systematically over represent journals from European and North American institutions, regardless of the language in which articles are written. This explains why, of the 43 included articles, only 6 feature Latin American samples (5 multinational and 1 from Colombia), compared to Europe (62%) and North America (22%), evidencing a limitation that is not merely linguistic but structural, reflecting inequalities in indexed scientific production and granting lesser visibility to regional platforms. Furthermore, the open-access criterion, adopted to ensure full-text accessibility during screening and coding, as well as procedural transparency and replicability, may have compounded this geographical bias: many relevant journals from the Global South operate under subscription or mixed-access models, meaning that this criterion likely introduced an additional layer of exclusion beyond that already imposed by the indexing structure of WoS and Scopus. These constraints should be taken into account when interpreting the findings of this review. Future reviews should broaden the corpus by including subscription-based sources and regional databases to better capture the geographical diversity of global research on youth authoritarianism.

Third, the predominance of quantitative cross-sectional designs (85%) restricts understanding of causal trajectories and longitudinal dynamics of youth authoritarian manifestations. Although 4 longitudinal studies and 2 mixed-methods studies were included, the absence of meta-analysis prevents the quantification of effect sizes and heterogeneity between thematic categories.

Finally, the 2015–2025 period excluded literature and publications prior to 2015, which could contextualise historical trends in youth authoritarianism. These methodological restrictions suggest caution in the generalization and the need for complementary reviews with in-depth qualitative approaches and greater geographical diversity.

## 6 Conclusions

This article identified areas of manifestation of authoritarianism among young people and analyzed their implications for democratic social life, based on recent research (2015–2025). The systematic literature review followed the quality criteria of the PRISMA statement (Yepes-Nuñez et al., 2021).

Based primarily on evidence from European and North American contexts, this systematic review suggests that authoritarianism in youth social life over the last 10 years may manifest through at least four interconnected levels or manifestations: social dominance, traditionalism, sexism and fundamentalism, mental health and parenting styles, and contextual and cultural perspectives. These are not isolated themes, but analytically distinct expressions of a common authoritarian continuum spanning psychological, ideological, relational, and contextual dimensions. These areas account for a reconfiguration of hierarchies in young people's social life, violence, and democratic relationships in contemporary global contexts.

The main findings show that social dominance articulates intergroup prejudices (racism, xenophobia), support for strong leadership, and symbolic/physical violence, legitimizing hierarchies in response to perceived threats and inequality. Traditionalism, sexism, and fundamentalism operate as normative matrices sustaining gender/sexual subordination and ethnic prejudices, integrating traditional body norms into authoritarian repertoires. Authoritarian socialization impacts mental health (emotional dysregulation, infantilisation) and prosociality with variable contextual effects, configuring rigid relational trajectories. Finally, democratic erosion, digital polarization, and perceived threats complicate youth authoritarianism, fostering both reproduction (technocracy, online uniformity) and forms of resistance (digital activism, climate concerns).

These results complement classical understandings of authoritarianism (Adorno et al., 1950; Altemeyer, 1981; Arendt, 1996; Weber, 1964), positioning it as a relational and situated phenomenon, not merely individual, threatening democratic coexistence through the normalization of control, exclusion, and everyday verticality. It must be noted, however, that these conclusions draw primarily on evidence from European and North American contexts (Europe 51%, North America 19%). Their applicability to other regions should be treated with caution until comparative research incorporating Global South perspectives is available. Likewise, future research should integrate longitudinal qualitative approaches to understand authoritarian manifestation dynamics in contexts of democratic erosion.

Finally, this study contributes to strengthening the development of preventive educational policies addressing intergroup dominance, rigid family socialization, and critical digital literacy, enhancing democratic resilience against institutional ruptures in contemporary democratic systems.

## Data Availability

The original contributions presented in the study are included in the article/Supplementary material, further inquiries can be directed to the corresponding author/s.

## References

[B1] Abbott P. Teti A. Sapsford R. (2020). The tide that failed to rise: Young people's politics and social values in and after the Arab uprisings. Mediterr. Polit. 25, 1–25. doi: 10.1080/13629395.2018.1482124

[B2] Adorno T. W. Frenkel-Brunswik E. Levinson D. Sanford R. N. (1950). The Authoritarian Personality. New York, NH: Harper & Brothers. Available online at: https://psycnet.apa.org/record/1950-05796-000

[B3] Adorno T. W. Frenkel-Brunswik E. Levinson D. J. Sanford R. N. (2006). La personalidad autoritaria (prefacio, introducción y conclusiones). Empiria Rev. Metodol. Cienc. Soc. 12, 155–200. doi: 10.5944/empiria.12.2006.1144

[B4] Agudelo-Hernández F. Vélez-Botero H. Guapacha-Montoya M. (2025). Human rights engagement, stigma and attitudes towards mental health among Colombian social work and medical students. Adv. Health Sci. Educ. 30, 1085–1100. doi: 10.1007/s10459-024-10377-5 PMC12390873 39503876

[B5] Aguilar-Forero N. (2022). Memoria y juvenicidio en el estallido social de Colombia (2021). Rev. Latinoam. Cienc. Soc. Niñez Juv. 20, 476–500. doi: 10.11600/rlcsnj.20.3.5492

[B6] Altemeyer R. (1981). Right-wing Authoritarianism. Winnipeg: Univ. of Manitoba press.

[B7] Alvarez S. Navarrete A. (2019). Cronología del movimiento feminista en Chile 2006–2016. Rev. Estud. Fem. 27:e54709. doi: 10.1590/1806-9584-2019v27n354709

[B8] Arendt H. (1996). Entre El Pasado y El Futuro. Ocho Ejercicios Sobre la Reflexión Polí*tica*. Barcelona: Ediciones Península.

[B9] Aromataris E. Fernandez R. Godfrey C. Holly C. Kahlil H. Tungpunkom P. (2015). Summarizing systematic reviews: methodological development, conduct and reporting of an umbrella review approach. Int. J. Evid. Based Healthc. 13, 132–140. doi: 10.1097/XEB.0000000000000055 26360830

[B10] Bardin L. (1991). Análisis de Contenido. Madrid: Ediciones Akal.

[B11] Bélanger J. Moyano M. Muhammad H. Richardson L. Lafrenière M. McCaffery P. . (2019). Radicalization leading to violence: a test of the 3N model. Front. Psychiatry 10:42. doi: 10.3389/fpsyt.2019.00042 30853917 PMC6396731

[B12] Benbenaste N. Delfino G. Vitale N. (2006). La contribución de la psicología al concepto de poder. Univ. Psychol. 5, 351–360.

[B13] Benjamin S. Koirikivi P. Salonen V. Gearon L. Kuusisto A. (2024). Safeguarding social justice and equality: exploring finnish youths'‘intergroup mindsets' as a novel approach in the prevention of radicalization and extremism through education. Educ. Citizsh. Soc. Justice 19, 292–312. doi: 10.1177/17461979221135845

[B14] Bobba B. Branje S. Crocetti E. (2024). Parents' and classmates' influences on adolescents' ethnic prejudice: a longitudinal multi-informant study. Child Dev. 95, 1522–1538. doi: 10.1111/cdev.14087 38456479

[B15] Borsuk I. Levin P. (2021). Social coexistence and violence during Turkey's authoritarian transition. Southeast Eur. Black Sea Stud. 21, 175–187. doi: 10.1080/14683857.2021.1909292

[B16] Cárdenas M. Parra L. (2010). Adaptación y validación de la versión abreviada de la escala de autoritarismos de derechas (RWA) en una muestra chilena. Rev. Psicol. 19, 61–79. doi: 10.5354/0719-0581.2010.17098

[B17] Carlo G. White R. Streit C. Knight G. Zeiders K. (2018). Longitudinal relations among parenting styles, prosocial behaviors, and academic outcomes in US Mexican adolescents. Child Dev. 89, 577–592. doi: 10.1111/cdev.12761 28213904 PMC5562534

[B18] Chiru M. Enyedi Z. (2022). Who wants technocrats? A comparative study of citizen attitudes in nine young and consolidated democracies. Br. J. Politics Int. Relation 24, 95–112. doi: 10.1177/13691481211018311

[B19] Cinquegrana V. Marini M. Galdi S. (2022). From endorsement of ambivalent sexism to psychological IPV victimization: the role of attitudes supportive of IPV, legitimating myths of IPV, and acceptance of psychological aggression. Front. Psychol. 13:922814. doi: 10.3389/fpsyg.2022.922814 35874380 PMC9301201

[B20] Conway L. Zubrod A. Chan L. McFarland J. Van de Vliert E. (2023). Is the myth of left-wing authoritarianism itself a myth?. Front. Psychol. 13:1041391. doi: 10.3389/fpsyg.2022.1041391 36846476 PMC9944136

[B21] Costello T. Bowes S. Stevens S. Waldman I. Tasimi A. Lilienfeld S. (2022). Clarifying the structure and nature of left-wing authoritarianism. J. Personal. Soc. Psychol. 122, 135. doi: 10.1037/pspp0000341 34383522

[B22] Costello T. Patrick C. J. (2023). Development and initial validation of two brief measures of left-wing authoritarianism: a machine learning approach. J. Pers. Assess. 105, 187–202. doi: 10.1080/00223891.2022.2081809 35767681

[B23] Da Costa Silva K. Torres A. Álvaro Estramiana J. Garrido Luque A. (2024). Discrimination against suspected Islamic terrorists: nationality, right-wing authoritarianism, and perceived threat as predictors of support for torture. Int. J. Psychol. 59, 859–870. doi: 10.1002/ijop.13150 38852955

[B24] Daldrop C. Buengeler C. Homan A. (2023). An intersectional lens on young leaders: bias toward young women and young men in leadership positions. Front. Psychol. 14:1204547. doi: 10.3389/fpsyg.2023.1204547 37663338 PMC10468608

[B25] Daldrop C. Homan A. Buengeler C. (2025). Too young to lead? Role incongruity explains age bias against young leaders. Leadersh. Q. 36:101878. doi: 10.1016/j.leaqua.2025.101878

[B26] Danilova Y. Danilova M. Troshikhina E. (2023). Psychosocial development in adolescents in condition of family deprivation. Psychiatr. Psychother. Clin. Psychol. 14. doi: 10.34883/PI.2023.14.2.007

[B27] Dávila M. Díaz-Romero P. Ensignia J. Espinoza O. Frigolett H. Gerber E. . (2020). La Demanda Ciudadana Por Una Nueva Democracia. Chile y El 18/O. Barómetro, 16. Santiago: Sur Ediciones.

[B28] Denzin N. (2017). Critical qualitative inquiry. Qual. Inq. 23, 8–16. doi: 10.1177/1077800416681864

[B29] Derado A. Dergić V. Medugorac V. (2016). Croatian youth and populism: a mixed methods analysis of the populism “breeding ground” among the youth in the City of Zagreb. Rev. Sociol. 46, 141–173. doi: 10.5613/rzs.46.2.2

[B30] Deverson S. Delfabbro P. Georgiou N. (2025). The moderating role of psychological distress in the relationship between postmodernism and left-wing authoritarianism. Appl. Cogn. Psychol. 39:e70021. doi: 10.1002/acp.70021

[B31] Disi Pavlic R. Medel R. Bargsted M. Somma N. (2025). Justification of violence, ideological preferences, and exposure to protests: causal evidence from the 2019 Chilean social unrest. Social Forces, soaf 102. doi: 10.1093/sf/soaf102

[B32] Duckitt J. (2001). “A dual-process cognitive-motivational theory of ideology and prejudice,” in Advances in Experimental Social Psychology (Academic Press), (Vol 33), 41–113. doi: 10.1016/S0065-2601(01)80004-6

[B33] Duckitt J. Bizumic B. Krauss S. Heled E. (2010). A tripartite approach to right-wing authoritarianism: the authoritarianism-conservatism-traditionalism model. Pol. Psych. 31, 685–715. doi: 10.1111/j.1467-9221.2010.00781.x

[B34] Epstein R. Bock S. Drew M. Scandalis Z. (2023). Infantilization across the life span: a large-scale internet study suggests that emotional abuse is especially damaging. Motiv. Emot. 47, 137–163. doi: 10.1007/s11031-022-09989-4

[B35] Etchezahar E. Barreiro A. Albalá Genol M. Maldonado A. (2022). Assessment of social justice dimensions in young adults: the contribution of the belief in a just world and social dominance orientation upon its rising. Front. Psychol. 13:997423. doi: 10.3389/fpsyg.2022.997423 36405221 PMC9670803

[B36] Gadamer H. (2004). Il Problema Della Coscienza Storica. Napoli: Guida Editori.

[B37] Ghafar A. (2021). Causes and consequences of inequality in Egypt. Muslim World 111, 5–26. doi: 10.1111/muwo.12370

[B38] González-Fuentes J. Moreno-Manso J. Guerrero-Molina M. (2024). Empatía y personalidad como variables predictoras de la homofobia sutil y manifiesta. Psychology 15, 1–13. doi: 10.5093/cc2024a11

[B39] Grindal M. Haltinner K. (2023). White racial identity and its link to support for far-right groups: a test of a social psychological model. Soc. Sci. 12:369. doi: 10.3390/socsci12070369

[B40] Guzmán-Concha C. (2023). Legacies of authoritarianism and elite responses to social unrest. The estallidos sociales in Peru and Chile. Sociológica 17, 105–117. doi: 10.6092/issn.1971-8853/16966

[B41] Hannover B. Gubernath J. Schultze M. Zander L. (2018). Religiosity, religious fundamentalism, and ambivalent sexism toward girls and women among adolescents and young adults living in Germany. Front. Psychol. 9:2399. doi: 10.3389/fpsyg.2018.02399 30559697 PMC6286999

[B42] Herzog B. (2021). Authoritarianism as pathology of recognition: the sociological substance and actuality of the authoritarian personality. Hum. Soc. Sci. Commun. 8, 1–9. doi: 10.1057/s41599-021-00819-5

[B43] Ilmarinen V. Sortheix F. Lönnqvist J. (2021). Consistency and variation in the associations between refugee and environmental attitudes in European mass publics. J. Environ. Psychol. 73:101540. doi: 10.1016/j.jenvp.2020.101540

[B44] Ipar E. Weglein L. Cuesta M. (2024). Discursos De Odio: Una Alarma Para la Vida Democrática. Buenos Aires: UNSAM edita.

[B45] Jylhä K. Rydgren J. Strimling P. (2022). Xenophobia among radical and mainstream right-wing party voters: prevalence, correlates and influence on party support. Ethn. Racial Stud. 45, 261–286. doi: 10.1080/01419870.2022.2061866

[B46] Karasavva V. Stewart J. Reynolds J. Forth A. (2025). The aggrieved entitlement scale: a new measure for an old problem. J. Interpers. Violence 40, 2906–2930. doi: 10.1177/08862605241280973 39344468 PMC12048742

[B47] Konopka K. Prusik M. Szulawski M. (2020). Two sexes, two genders only: measuring attitudes toward transgender individuals in Poland. Sex Roles. 82, 600–621. doi: 10.1007/s11199-019-01071-7

[B48] Krämer M. (2020). Neotraditional authority contested: the corporatization of tradition and the quest for democracy in the topnaar traditional authority, Namibia. Africa 90, 318–338. doi: 10.1017/S0001972019001062

[B49] Latinobarómetro (2024). Informe 2024. Santiago de Chile: Corporación Latinobarómetro. Available online at: https://www.latinobarometro.org/lat.jsp?Idioma=0

[B50] Lesgart C. (2020). Authoritarianism. History and problems of a fundamental contemporary concept. Perf. Latinoam. 28, 349–371. doi: 10.18504/pl2855-014-2020

[B51] Levitsky S. Ziblatt D. (2018). Cómo Mueren Las Democracias. Barcelona: Ariel.

[B52] Lockwood C. Munn Z. Porritt K. (2015). Qualitative research synthesis: methodological guidance for systematic reviewers utilizing meta-aggregation. Int. J. Evid. Based Healthc. 13, 179–187. doi: 10.1097/XEB.0000000000000062 26262565

[B53] López R. Pereyra G. (2021). Régimen, derechos fundamentales y sociales en Latinoamérica, 2019. Telos 23, 51–63. doi: 10.36390/telos231.05

[B54] López-Hornickel N. Carrasco D. Lay S. Treviño E. (2024). It is not just your opinion. gender equity endorsement of Latin American students and their peers at school. Large-Scale Assess. Educ. 12:45. doi: 10.1186/s40536-024-00235-6

[B55] Magack E. David A. Ogodo H. (2025). “Digital natives in Aktion: erforschung des digitalen verhaltens der generation Z und ihrer gesellschaftlichen auswirkungen in Kenia,”. in *Generation Z International*: A*nsprache und Rekrutierung Junger Talente*, eds. C. Kochhan and G. Bolduan (Wiesbaden: Springer Gabler). doi: 10.1007/978-3-658-49350-9_9

[B56] Majtényi B. Ryder A. (2025). Hungarian academia in a deep state. Eur. Rev. 33, 1–17. doi: 10.1017/S106279872500016X

[B57] McArthur A. Cooper A. Edwards D. Klugarova J. Yan H. Barber B. V. . (2025). Textual evidence systematic reviews series paper 3: critical appraisal of evidence from narrative, opinion, and policy. JBI Evid. Synthesis 23, 833–839. doi: 10.11124/JBIES-24-00293 39905824

[B58] Méndez I. Ruiz-Esteban C. López-García J. (2017). Risk and protective factors associated to peer school victimization. Front. Psychol. 8:441. doi: 10.3389/fpsyg.2017.00441 28382016 PMC5360713

[B59] Monsivais-Carrillo A. (2020). La indiferencia hacia la democracia en América Latina. Íconos 66, 151–171. doi: 10.17141/iconos.66.2020.3469

[B60] Moreno A. Lagos M. (2024). La medición del autoritarismo en América Latina: retos para la ciencia política. Rev. Mex. Cienc. Polít. Soc. 69, 143–164. doi: 10.22201/fcpys.2448492xe.2024.251.87750

[B61] Moreno-Jiménez M. Rodríguez M. Martín M. (2013). Construction and validation of the community and socio-political participation scale (SCAP). Spanish J. Psychol. 16:E42. doi: 10.1017/sjp.2013.48 23866238

[B62] Moyano M. Lobato R. Blaya-Burgo M. Arnal N. Cuadrado E. Mateu D. . (2022). Preventing violent extremism in youth through sports: an intervention from the 3N model. Psychol. Sport Exercise. 63:102283. doi: 10.1016/j.psychsport.2022.102283

[B63] Off G. Charron N. Alexander A. (2022). Who perceives women's rights as threatening to men and boys? Explaining modern sexism among young men in Europe. Front. Polit. Sci. 84. doi: 10.3389/fpos.2022.909811

[B64] Osborne D. Costello T. Duckitt S. (2023). The psychological causes and societal consequences of authoritarianism. Nat. Rev. Psychol. 2, 220–232. doi: 10.1038/s44159-023-00161-4 37056296 PMC9983523

[B65] Page M. McKenzie J. Bossuyt P. Boutron I. Hoffmann T. Mulrow C. . (2021). The PRISMA 2020 statement: an updated guideline for reporting systematic reviews. BMJ 372:n71. doi: 10.1136/bmj.n71 33782057 PMC8005924

[B66] Parent M. Silva K. (2018). Critical consciousness moderates the relationship between transphobia and “bathroom bill” voting. J. Counsel. Psychol. 65:403. doi: 10.1037/cou0000270 PMC10460521 29999367

[B67] Parra E. Zorro Y. (2020). Representaciones sociales de los jóvenes frente a la pandemia COVID-19. Exp. Investig. Signif. 6:11–11.

[B68] Pauwels L. Williamson H. (2024). Explaining prejudicial attitudes and bias-motivated aggression in Belgium: a comparison of individual-level theoretical models. Eur. J. Crim. Pol. Res. 30, 109–134. doi: 10.1007/s10610-022-09529-3

[B69] Pinquart M. Lauk J. (2025). Associations of parenting styles with substance use in the offspring—A systematic review and meta-analysis. Drug Alcohol Rev. 44, 133–143. doi: 10.1111/dar.13961 39397326 PMC11743216

[B70] Pirzada S. Valadez E. (2025). Sex differences in the associations among parenting, socioeconomic status, and error monitoring among adolescents. Dev. Psychobiol. 67:e70023. doi: 10.1002/dev.70023 39935245 PMC11814918

[B71] Prati G. Tzankova I. Albanesi C. Cicognani E. (2022). Longitudinal predictors of perceived climate change importance and worry among Italyn youths: a machine learning approach. Sustainability 14:15716. doi: 10.3390/su142315716

[B72] Ramírez M. Wood R. (2023). Authoritarian opposition? Authoritarian disposition and resistance to public health mitigation strategies during COVID-19. *Pol. Res*. Q. 77, 239–254. doi: 10.1177/10659129231204234

[B73] Rivera-Aguilera G. Imas M. Jiménez-Díaz L. (2021). Jóvenes, multitud y estallido social en Chile. Rev. Latinoam. Cienc. Soc. Niñez Juv. 19, 230–252. doi: 10.11600/rlcsnj.19.2.4543

[B74] Rollero C. Bergagna E. Tartaglia S. (2021). What is violence? The role of sexism and social dominance orientation in recognizing violence against women. *J. Interpers*. Violence 36, NP11349–NP11366. doi: 10.1177/0886260519888525 31744362

[B75] Rose J. (2025). Meta economics: generating moral economies. Int. Rev. Appl. Econom. 39, 175–180. doi: 10.1080/02692171.2025.2473911

[B76] Russo S. Roccato M. Merlone U. (2020). Actual threat, perceived threat, and authoritarianism: an experimental study. Spanish J. Psychol. 23:e3. doi: 10.1017/SJP.2020.7 32519639

[B77] Sánchez-Serrano S. Pedraza-Navarro I. Donoso-González M. (2022). ¿Cómo hacer una revisión sistemática siguiendo el protocolo PRISMA? Usos y estrategias fundamentales para su aplicación en el ámbito educativo a través de un caso práctico. Bordón Rev. Pedagog. 74, 51–66. doi: 10.13042/Bordon.2022.95090

[B78] Saroglou V. Clobert M. Cohen A. Johnson K. Ladd K. Brandt P.Y. . (2022). Fundamentalism as dogmatic belief, moral rigorism, and strong groupness across cultures: dimensionality, underlying components, and related interreligious prejudice. Psychol. Relig. Spiritual. 14:558. doi: 10.1037/rel0000339

[B79] Schäfer A. (2022). Cultural backlash? How (not) to explain the rise of authoritarian populism. Br. J. Pol. Sci. 52, 1977–1993. doi: 10.1017/S0007123421000363

[B80] Shaminov R. Bocharova E. Nevsky E. V. Suzdaltsev N. Akaemova U. (2023). The role of attitudes towards authoritarianism and social trust in the manifestations of civic and on-line activity. Exp. Psychol. 16:102. doi: 10.17759/exppsy.2023160207

[B81] Slone M. Shoshani A. (2017). Children affected by war and armed conflict: parental protective factors and resistance to mental health symptoms. Front. Psychol. 8:1397. doi: 10.3389/fpsyg.2017.01397 28878705 PMC5572511

[B82] Sochos A. (2021). Authoritarianism, trauma, and insecure bonds during the Greek economic crisis. Curr. Psychol. 40, 1923–1935. doi: 10.1007/s12144-018-0111-5

[B83] Sprong S. Jetten J. Wang Z. Peters K. Mols F. Verkuyten M. . (2019). “Our country needs a strong leader right now”: economic inequality enhances the wish for a strong leader. Psychol. Sci. 30, 1625–1637. doi: 10.1177/0956797619875472 31566081

[B84] Ştefănel A. (2025). Authoritarian drift and social media's impact on Romanian youth during the 2024 European elections. Media Commun. 13. doi: 10.17645/mac.10617

[B85] Stiglitz J. E. (2010). El Malestar en la Globalización. Madrid: Taurus.

[B86] Strauss A. (1987). Qualitative Analysis for Social Scientists. Cambridge: Cambridge university press. doi: 10.1017/CBO9780511557842

[B87] Taufik A. Farnanda. (2019). Fundamentalism among acehnese youth: acehnese university students'attitudes on religious fundamentalism, right-wing authoritarianism, and militia sentiments. Hum. Soc. Sci. Rev. 6, 1–8. doi: 10.18510/hssr.2018.631

[B88] Tavares F. da Silva J. (2021). Neoliberalismo como autoritarismo no brasil contemporâneo: Declínio democrático e perecimento constitucional em nome do mercado, da ordem e da família. Bol. Goiano Geogr. 41:15. doi: 10.5216/bgg.v41.70041

[B89] Taylor S. Bogdan R. (1987). Introducción a Los Métodos Cualitativos de Investigación. Barcelona: Paidós.

[B90] Torres-Vega L. C. Ruiz J. Moya M. (2021). Dangerous worldview and perceived sociopolitical control: two mechanisms to understand trust in authoritarian political leaders in economically threatening contexts. Front. Psychol. 12:603116. doi: 10.3389/fpsyg.2021.603116 33841238 PMC8027088

[B91] Travaglino G. Friehs M. Kotzur P. Abrams D. (2023). Investigating the social embeddedness of criminal groups: longitudinal associations between masculine honour and legitimizing attitudes towards the Camorra. Eur. J. Soc. Psychol. 53, 612–622. doi: 10.1002/ejsp.2926

[B92] Troncoso-Tejada G. Gálvez-Nieto J. L. Norambuena-Paredes I. Galván-Cabello M. Casas D. G. (2025a). Psychometric study and validation of an abbreviated version of the left-wing authoritarianism scale (LWA-9) in Chilean university students. Front. Psychol. 16:1627540. doi: 10.3389/fpsyg.2025.1627540 40949355 PMC12426141

[B93] Troncoso-Tejada G. Quintano-Méndez F. Norambuena-Paredes I. (2025b). Confianza perdida: ¿autoritarismo en el horizonte? Juventud chilena y la participación política en un contexto de crisis democrática. Desde el Sur 17:e0095. doi: 10.21142/DES-1704-2025-0095

[B94] Valencia R. (2024). La ola autoritaria y el extremismo en el mundo durante la pandemia de COVID-19. Context. Latinoam. 2, 71–82. doi: 10.32870/cl.v2i31.8064

[B95] Verkuyten M. Yogeeswaran K. (2017). The social psychology of intergroup toleration: aroadmap for theory and research. Personal. Soc. Psychol. Rev. 21, 72–96. doi: 10.1177/1088868316640974 27069001

[B96] Voces C. López M. (2022). Pandemia y actitudes hacia el sistema político.¿ Han cambiado las preferencias por la democracia y el autoritarismo? Methaodos. Rev. Ciencias Sociales 10, 177–192. doi: 10.17502/mrcs.v10i2.549

[B97] Weber L. Bauer L. Führer A. (2025). Hierarchy hurts: a comparative cross-sectional analysis of empathy and its determinants in medical, midwifery, and nursing students. BMC Med. Educ. 25:1076. doi: 10.1186/s12909-025-07683-w 40682009 PMC12273016

[B98] Weber M. (1964). Economí*a y Sociedad: Esbozo de Sociolog*í*a Comprensiva*. México: Fondo de Cultura Económica..

[B99] Yavuz M. (2018). A framework for understanding the intra-Islamist conflict between the AK party and the Gülen movement. Politics, Relig. Ideol. 19, 11–32. doi: 10.1080/21567689.2018.1453247

[B100] Yepes-Nuñez J. Urrutia G. Romero García M. Alonso-Fernandez S. (2021). The PRISMA 2020 statement: an updated guideline for reporting systematic reviews declaración PRISMA 2020: una guía actualizada para la publicación de revisiones sistemáticas. Rev. Esp. Cardiol. 74, 790–799. 34446261 10.1016/j.rec.2021.07.010

[B101] Yerofeyeva V. Wang P. Yang Y. Serobyan A. Grigoryan A. Nartova-Bochaver S. (2024). Shimmering emerging adulthood: in search of the invariant IDEA model for collectivistic countries. Front. Psychol. 15:1349375. doi: 10.3389/fpsyg.2024.1349375 38650904 PMC11034521

[B102] Zhang J. Mollandsøy A. Nornes C. Erevik E. Pallesen S. (2025). Predicting hostility towards women: incel-related factors in a general sample of men. Scand. J. Psychol. 66, 35–46. doi: 10.1111/sjop.13062 39104169 PMC11735252

