# Supplementary material for: Authoritarianism in youth social life: systematic literature review (2015–2025)
Source: Front Psychol. 2026 May 22;17:1794108. doi: 10.3389/fpsyg.2026.1794108 (PMC13238435; doi:10.3389/fpsyg.2026.1794108)
Supplement: Supplementary file 2 [file Data_Sheet_2.docx]

| **Supplementary material 2**  **Quality assessment according to the Joanna Briggs Institute Critical Appraisal Tools (JBI)** | | | | |
| --- | --- | --- | --- | --- |
| **Study Number** | **Author** | **Type of study** | **JBI Score** | **Quality rating** |
| 1 | Benjamin, S.; Koirikivi, P.; Salonen, V.; Gearon, L.; Kuusisto, A. | Cross sectional studies | 8/8=100% | High |
| 2 | Pauwels, L.J.R.; Williamson, H. | Cross sectional studies | 8/8=100% | High |
| 3 | Grindal, M.; Haltinner, K. | Cross sectional studies | 8/8=100% | High |
| 4 | Travaglino, G.A.; Friehs, M.-T.; Kotzur, P.F.; Abrams, D. | Cross sectional studies | 7/8=88% | High |
| 5 | Daldrop, C.; Buengeler, C.; Homan, A.C. | Cross sectional studies | 8/8=100% | High |
| 6 | Etchezahar, E.; Barreiro, A.; Albalá Genol, M.Á.; Maldonado, A.F. | Cross sectional studies | 7/8=88% | High |
| 7 | Sprong, S., et al. | Cross sectional studies | 8/8=100% | High |
| 8 | Derado, A.; Dergić, V.; Medugorac, V. | Cross sectional studies | 6/8= 75% | High |
|  |  | Qualitative research | 8/10= 80% | High |
| 9 | Ilmarinen, VJ; Sortheix, FM; Lönnqvist, JE | Cross sectional studies | 5/8=63% | Moderate |
| 10 | Jylhä, KM; Rydgren, J; Strimling, P | Cross sectional studies | 6/8= 75% | High |
| 11 | Weber, L.M.; Bauer, L.; Führer, A. | Cross sectional studies | 8/8=100% | High |
| 12 | Daldrop, C; Homan, AC; Buengeler, C | Cross sectional studies | 8/8=100% | High |
| 13 | Rollero, C.; Bergagna, E.; Tartaglia, S. | Cross sectional studies | 7/8=88% | High |
| 14 | Saroglou, V., et al. | Cross sectional studies | 7/8=88% | High |
| 15 | Hannover, B.; Gubernath, J.; Schultze, M.; Zander, L. | Cross sectional studies | 8/8=100% | High |
| 16 | Parent, M.C.; Silva, K. | Cross sectional studies | 8/8=100% | High |
| 17 | Taufik, A.A. & Farnanda | Cross sectional studies | 6/8= 75% | High |
| 18 | Konopka, K; Prusik, M; Szulawski, M | Cross sectional studies | 8/8=100% | High |
| 19 | Off, G; Charron, N; Alexander, A | Cross sectional studies | 7/8=88% | High |
| 20 | Zhang, JW; Mollandsoy, AB; Nornes, C; Erevik, EK; Pallesen, S | Cross sectional studies | 8/8=100% | High |
| 21 | Karasavva, V.; Stewart, J.; Reynolds, J.; Forth, A. | Cross sectional studies | 8/8=100% | High |
| 22 | Cinquegrana, V; Marini, M; Galdi, S | Cross sectional studies | 8/8=100% | High |
| 23 | González-Fuentes, JA; Moreno-Manso, JM; Guerrero-Molina, M | Cross sectional studies | 7/8=88% | High |
| 24 | Danilova, Y.; Danilova, M.; Troshikhina, E. | Cross sectional studies | 6/8= 75% | High |
| 25 | Carlo, G.; White, R.M.B.; Streit, C.; Knight, G.P.; Zeiders, K.H. | Cross sectional studies | 8/8=100% | High |
| 26 | Epstein, R; Bock, SD; Drew, MJ; Scandalis, Z | Cross sectional studies | 5/8= 63% | Moderate |
| 27 | Bobba, B; Branje, S; Crocetti, E | Cross sectional studies | 8/8=100% | High |
| 28 | Agudelo Hernández, F.; Vélez-Botero, H.; Guapacha-Montoya, M. | Cross sectional studies | 6/8= 75% | High |
| 29 | Pirzada, S.; Valadez, E.A. | Cross sectional studies | 8/8=100% | High |
| 30 | Pinquart, M.; Lauk, J. | Systematic reviews and research syntheses | 11/11= 100% | High |
| 31 | Slone, M.; Shoshani, A. | Cross sectional studies | 8/8=100% | High |
| 32 | Prati, G.; Tzankova, I.; Albanesi, C.; Cicognani, E. | Cross sectional studies | 7/8=88% | High |
| 33 | Chiru, M.; Enyedi, Z. | Cross sectional studies | 7/8=88% | High |
| 34 | Russo, S.; Roccato, M.; Merlone, U. | Cross sectional studies | 8/8=100% | High |
| 35 | Abbott, P; Teti, A; Sapsford, R | Cross sectional studies | 6/8= 75% | High |
| 36 | Shamionov, RM; Bocharova, EE; Nevskiy, EV; Suzdaltcev, NV; Akayomova, UA | Cross sectional studies | 8/8=100% | High |
| 37 | Voces, C; Caínzos, M | Cross sectional studies | 7/8=88% | High |
| 38 | Méndez, I; Ruiz-Esteban, C; López-García, JJ | Cross sectional studies | 8/8=100% | High |
| 39 | Majtényi, B.; Ryder, A. | Textual evidence: narrative | 6/6= 100% | High |
| 40 | Stefanel, A. | Cross sectional studies | 7/8=88% | High |
| 41 | López-Hornickel, N.; Carrasco, D.; Lay, S.; Treviño, E. | Cross sectional studies | 8/8=100% | High |
| 42 | Krämer, M. | Qualitative research | 10/10=100% | High |
| 43 | Schäfer, A | Textual evidence: narrative | 4/6=67% | Moderate |
