# Supplementary material for: Authoritarianism in youth social life: systematic literature review (2015–2025)
Source: Front Psychol. 2026 May 22;17:1794108. doi: 10.3389/fpsyg.2026.1794108 (PMC13238435; doi:10.3389/fpsyg.2026.1794108)
Supplement: Supplementary file 3 [file Table_1.docx]

**Supplementary Table 1.** Bibliographic database search strategies.

| **Database** | **Search terms** | **Results (number of articles, n)** |
| --- | --- | --- |
| Web of Science | TS=(( authoritarianism OR "social dominance orientation" OR "authoritarian ideology" OR "authoritarian attitude" OR "authoritarian beliefs" OR "authoritarian conviction" OR "antidemocratic ideology" OR "antidemocratic attitude" OR "antidemocratic beliefs" OR "antidemocratic conviction" OR "illiberal ideology" OR "illiberal attitude" OR "illiberal beliefs" OR "illiberal conviction" OR "nondemocratic ideology" OR "nondemocratic attitude" OR "nondemocratic beliefs" OR "nondemocratic conviction" OR "fascist ideology" OR "fascist attitude" OR "fascist beliefs" OR "fascist conviction") AND ("young people” OR youth OR young OR “young population” OR adolescents OR teen OR teenagers) AND (perception OR assessment OR appreciation OR attitude)) AND PY=(2015 OR 2016 OR 2017 OR 2017 OR 2018 OR 2019 OR 2020 OR 2021 OR 2022 OR 2023 OR 2024 OR 2025) | n=412  final: n=15 |
| Scopus | TITLE-ABS-KEY(( authoritarianism OR "social dominance orientation" OR "authoritarian ideology" OR "authoritarian attitude" OR "authoritarian beliefs" OR "authoritarian conviction" OR "antidemocratic ideology" OR "antidemocratic attitude" OR "antidemocratic beliefs" OR "antidemocratic conviction" OR "illiberal ideology" OR "illiberal attitude" OR "illiberal beliefs" OR "illiberal conviction" OR "nondemocratic ideology" OR "nondemocratic attitude" OR "nondemocratic beliefs" OR "nondemocratic conviction" OR "fascist ideology" OR "fascist attitude" OR "fascist beliefs" OR "fascist conviction" ) AND ( "young people” OR youth OR young OR “young population” OR adolescents OR teen OR teenagers) AND ( perception OR assessment OR appreciation OR attitude)) AND DOCTYPE ( ar OR re ) AND PUBYEAR > 2015 | n=269  final: n=28 |
| Total | | n=43 |
| Total after removing duplicates | | n=24 |

|  |
| --- |
